# Outcomes following intradetrusor onabotulinumtoxinA injections and sacral neuromodulation in older men

**DOI:** 10.1002/bco2.70168

**Published:** 2026-03-31

**Authors:** Leo D. Dreyfuss, Lufan Wang, Farnoosh Nik‐Ahd, Abigail Shatkin‐Margolis, Kenneth Covinsky, W. John Boscardin, Anne M. Suskind

**Affiliations:** ^1^ Department of Urology Weill Cornell Medical Center New York New York USA; ^2^ Department of Urology University of California San Francisco California USA; ^3^ Department of Obstetrics and Gynecology University of California San Francisco California USA; ^4^ Division of Geriatrics University of California San Francisco California USA; ^5^ Department of Epidemiology and Biostatistics University of California San Francisco California USA

**Keywords:** botox, elderly, frailty, OAB, SNM

## Abstract

**Objectives:**

This study aims understand the outcomes following intradetrusor onabotulinumtoxinA injections and sacral neuromodulation (SNM) in a national cohort of male Medicare beneficiaries with overactive bladder (OAB)

**Subjects and Methods:**

This is a retrospective cohort study of a 100% sample of fee‐for‐service male Medicare beneficiaries undergoing first‐time onabotulinumtoxinA injections or SNM test procedures from 2014 to 2016. The primary outcome was repeat onabotulinumtoxinA injections within 1 year of index injections and placement of implantable pulse generator (IPG) within 90 days of a SNM test procedure.

**Results:**

Overall, 11 286 male beneficiaries were included; 31.4% who underwent onabotulinumtoxinA injections had repeat injections within 1 year and 56.0% who underwent SNM test procedures had an IPG implant within 90 days. Repeat onabotulinumtoxinA injections were less likely among men who were ≥85 years (adjusted relative risk [aRR] 0.85, 0.75–0.96) versus ages 65–74. IPG implantation was more likely following staged versus percutaneous nerve evaluation (PNE; aRR 1.57, 1.50–1.64) and among beneficiaries in the lowest versus highest of socioeconomic quartile (aRR 1.10, 1.03–1.18).

**Conclusions:**

Rates of repeat treatments following index onabotulinumtoxinA injections among older men were lower than those previously reported among younger, predominantly female populations, whereas rates of IPG implantation were similar. Interestingly, frailty and comorbidity were not associated with these claims‐based treatment outcomes following either procedure, but certain non‐clinical variables were associated with IPG implantation following SNM. While these outcomes cannot distinguish symptom improvement from discontinuation because of lack of efficacy, adverse events or patient preference, they provide important insights into real‐world treatment patterns for a patient population which is under‐sampled in the OAB literature.

## 1 INTRODUCTION

Overactive bladder (OAB) affects up to 30% of men over the age of 65.^1^ Management of OAB in men can be additionally complicated by frequent co‐occurrence of bladder outlet obstruction (BOO) secondary to benign prostatic hyperplasia (BPH). These overlapping conditions can potentially lead to undertreatment or delay in treatment of OAB‐related symptoms.^2^ While treatment for OAB often starts with pharmacotherapy, older men who receive bladder‐specific medications tend to experience high rates of early medication discontinuation because of side effects and poor efficacy.^3^ For these men who are unable or unwilling to take bladder‐specific medications for OAB, clinicians may consider utilisation of minimally invasive therapies for OAB, such as onabotulinumtoxinA injections or sacral neuromodulation (SNM).^4^

However, despite the high burden of OAB among older men, little is published about the use of these minimally invasive OAB therapies specifically in this population. Landmark trials on these therapies tend to focus on younger, predominantly female subjects,^5^, ^6^ and small retrospective series suggest that men may experience lower efficacy and higher complication rates compared to females, possibly because of sex‐specific anatomical differences.^7^ As a result, clinicians must employ a trial‐and‐error approach based on clinical judgement/experience or prior studies based on altogether different patient populations being treated for OAB.^8^

To better understand outcomes of minimally invasive OAB therapies in older men, this study uses a 100% sample of fee‐for‐service male Medicare beneficiaries who received first‐time treatment from 2014 to 2016 to calculate rates of repeat onabotulinumtoxinA treatment following initial injection and progression from SNM test procedure (percutaneous nerve evaluation [PNE] or permanent tined lead placement [Stage 1]) to implantable pulse generator (IPG) placement. These findings will allow clinicians to better counsel older men on outcomes following minimally invasive OAB therapy.

## 2 SUBJECTS AND METHODS

### 2.1 Database and subjects

This is a population‐based retrospective cohort study of a 100% sample of fee‐for‐service male Medicare beneficiaries ages 66 or older in the United States who underwent first‐time onabotulinumtoxinA injections or SNM test procedures for OAB between 2014 and 2016. Beneficiaries were identified according to current procedural terminology (CPT‐4) codes from the Medicare Carrier file. Only procedure codes with associated International Classification of Disease (ICD) 9th and 10th edition diagnosis codes for OAB were included and were adapted from previously published work (Table S1).^9^ Beneficiaries with less than 1 year of continuous enrollment before the date of the index procedure, or those who died within 1 year of index procedure, were excluded. To identify first‐time procedures, beneficiaries who underwent onabotulinumtoxinA injections, SNM test procedures, IPG placement, or SNM explant or revision in the year prior to the study period (including 2013) were also excluded (Table S1). The study was deemed to be exempt by our institution's review board.

### 2.2 Outcome measures

The primary outcome for this study was defined according to the type of minimally invasive OAB therapy. The primary outcome following onabotulinumtoxinA injection was repeat treatments within 1 year, and, following SNM test procedures, the primary outcome was IPG placement within 90 days, consistent with prior work.^10^, ^11^ Beneficiaries who underwent PNE and had subsequent claims for Stage 1 and IPG placement on three different visits were classified as having progressed following Stage 1 procedure instead of PNE. Beneficiaries who underwent PNE followed by simultaneous claims for Stage 1 and IPG placement on the same visit were classified as having progressed following PNE. Beneficiaries with claims for Stage 1 and PNE on the same visit or those who underwent single‐stage procedures (test procedure and IPG placement on the same day) were excluded, as it is unclear what procedure was performed based on this combination of billing codes.

Secondary outcomes included 30‐day complications following index minimally invasive OAB therapy and rates of device explant or revision within 1‐year of IPG placement. Complications were identified using diagnosis codes consistent with prior studies.^12^ ‘Other infections’ included pyelonephritis, cystopyelitis, severe sepsis with septic shock and bacteraemia. Rates of 1‐year device explant or revision were calculated among the subset of beneficiaries who were alive 1‐year following the successful IPG placement.

### 2.3 Covariates

Demographic data including age group (65–74, 75–84, ≥85 years) and race (White, Black, Other) were obtained using the Master Beneficiary Summary file. Charlson Comorbidity Index (CCI) was calculated using ICD‐9 and ICD‐10 codes contained within the Medicare MedPAR, Outpatient and Carrier files as previously described.^13^ Frailty was measured using the Claims‐based Frailty Index (CFI), which has been described previously.^14^, ^15^ Socioeconomic status was measured using the Area Deprivation Index (ADI) scaled to 100, which represents the social deprivation percentile of a beneficiary's zip code. A higher ADI represents greater social deprivation (lower socioeconomic status) and has been shown to correlate with adverse health outcomes.^16^ Beneficiaries who underwent urodynamics were identified using CPT codes for urodynamics procedures, which included cystometrogram in the year prior to index procedure (Table S1) in accordance with prior work.^17^

### 2.4 Statistical analysis

Categorical and continuous variables were reported using chi‐squared test and analysis of variance (ANOVA), respectively. Given the large size of treatment groups, standard mean difference (SMD) was used to quantify the magnitude of difference for baseline characteristics between the onabotulinumtoxinA and SNM groups,^18^, ^19^ with an SMD of <0.2 representing a small difference (>85% distribution overlap) between groups and thus similar values. Corresponding *p*‐values that were statistically significant were denoted as such. Multivariable models were constructed using generalised linear regression models with log link, Poisson distribution and robust standard errors to identify predictors of treatment outcomes. Independent variables in the model included age, race, CCI, CFI, ADI, procedure year, pre‐procedure urodynamics and type of SNM procedure (Stage 1 vs. PNE), when applicable.

While the primary outcome following onabotulinumtoxinA injection was defined as repeat injection within 1 year of the index procedure, the cumulative incidence of repeat injections at 2 and 3 years was estimated using Kaplan–Meier analysis among the subset of beneficiaries who underwent index injection early enough in the study period to allow at least 2 or 3 years of continuous Medicare enrolment and survival. Kaplan–Meier analysis was also used to estimate 1‐year rates of SNM explant or revision among beneficiaries who underwent IPG placement. Per the Centers for Medicare and Medicaid Services Cell Suppression Policy, cells containing values between 1 and 10 were masked.^20^

## 3 RESULTS

A total of 11 286 male beneficiaries underwent minimally invasive therapy for OAB between 2014 and 2016, including 6166 (54.6%) who underwent onabotulinumtoxinA injections and 5120 (45.4%) who underwent SNM. Table 1 reports baseline demographics of the study cohort stratified by the type of minimally invasive OAB procedure. No clinically significant differences were observed between groups for age, CCI, CFI, ADI and pre‐procedure urodynamics procedures. The number of male beneficiaries who underwent onabotulinumtoxinA injections increased from 1600 in 2014 to 2520 in 2016, while the number of SNM test procedures decreased from 1852 procedures to 1627 procedures, respectively (*p* < 0.05, SMD = 0.240). Among male beneficiaries undergoing SNM test procedures, 4015 (78.4%) had PNEs versus a formal staged approach.

**TABLE 1:** Baseline characteristics of male Medicare beneficiaries who underwent onabotulinumtoxinA injections or sacral neuromodulation (SNM) test procedures, 2014–2016. (SMD = standard mean difference, SD = standard deviation).

Variable name	Total	OnabotulinumtoxinA	SNM	SMD^a^
	11 286 (100.0)	*n* = 6166 (54.6)	*N* = 5120 (45.4)	
Age in years				
65–74	4343 (38.5)	2266 (36.8)	2077 (40.6)	0.105^b^
75–84	5192 (46.0)	2851 (46.2)	2341 (45.7)	
≥ 85	1751 (15.5)	1049 (17.0)	702 (13.7)	
Mean ± SD	77.6 ± 6.7	77.9 ± 6.8	77.2 ± 6.5	0.106^b^
Race				
White	10 121 (89.7)	5517 (89.5)	4604 (89.9)	0.027
Black	662 (5.9)	359 (5.8)	303 (5.9)	
Other	503 (4.5)	290 (4.7)	213 (4.2)	
Charlson comorbidity index				
0	3780 (33.5)	1915 (31.1)	1865 (36.4)	0.150^b^
1	2073 (18.4)	1090 (17.7)	983 (19.2)	
2	1910 (16.9)	1082 (17.6)	828 (16.2)	
3	1254 (11.1)	709 (11.5)	545 (10.6)	
≥ 4	2269 (20.1)	1370 (22.2)	899 (17.6)	
Mean ± SD	2.0 ± 2.2	2.1 ± 2.2	1.8 ± 2.1	0.144^b^
Claims‐based frailty index				
Not frail (CFI < 0.15)	2518 (22.3)	1338 (21.7)	1180 (23.1)	0.066^b^
Prefrail (0.15 ≥ CFI < 0.25)	6417 (56.9)	3471 (56.3)	2946 (57.5)	
Mildly to severely frail (CFI ≥ 0.25)	2351 (20.8)	1357 (22.0)	994 (19.4)	
Mean ± SD	0.2 ± 0.1	0.2 ± 0.1	0.2 ± 0.1	0.070^b^
Area deprivation index national quartile				
Q1: 1 ≤ 29	2885 (25.6)	1739 (28.3)	1146 (22.4)	0.143^b^
Q2: 29 ≤ 48	2819 (25.0)	1515 (24.6)	1304 (25.5)	
Q3: 48 ≤ 66	2781 (24.7)	1488 (24.2)	1293 (25.3)	
Q4: ≥66	2784 (24.7)	1413 (23.0)	1371 (26.8)	
Procedure year				
2014	3452 (30.6)	1600 (26.0)	1852 (36.2)	0.240^b^
2015	3687 (32.7)	2046 (33.2)	1641 (32.1)	
2016	4147 (36.7)	2520 (40.9)	1627 (31.8)	
Urodynamics in year prior to procedure				
No	6705 (59.4)	3838 (62.2)	2867 (56.0)	0.127^b^
Yes	4581 (40.6)	2328 (37.8)	2253 (44.0)	
Type of SNM test procedures				
Percutaneous nerve evaluation (PNE)	4015 (78.4)	‐‐	4015 (78.4)	‐‐
Stage 1	1105 (21.6)	‐‐	1105 (21.6)	‐‐

^a^ SMD of <0.2 corresponds to >85% overlap between distributions, representing similarity between the two groups (not clinically significant).

^b^ Corresponding *p*‐value < 0.05.

Overall, 1788 (31.4%) of beneficiaries who underwent index onabotulinumtoxinA injections had a repeat injection within 1 year. Table 2 shows the multivariable model for repeat injections following index onabotulinumtoxinA procedure. Male beneficiaries who were age ≥85 years versus 65–74 (adjusted relative risk [aRR] 0.85, 95% confidence interval [CI] 0.75–0.96) were less likely to undergo repeat onabotulinumtoxinA injections within 1 year. Pre‐operative urodynamics testing in the year prior to index procedure was associated with a higher adjusted risk of repeat onabotulinumtoxinA injections (aRR 1.13, 95% CI 1.04–1.22). Race, CCI, CFI and ADI were not significantly associated with repeat onabotulinumtoxinA injections. While the primary outcome was defined as rates of repeat onabotulinumtoxinA injections within 1 year of the index procedure, Kaplan–Meier analysis was used to estimate rates of repeat injections in a subset of beneficiaries whose index procedure occurred early enough in the study period to allow at 2 or 3 years of continuous enrolment and survival after the index injection. Among these beneficiaries, the Kaplan–Meier‐derived cumulative incidence of repeat onabotulinumtoxinA injections increased from 31.4% at 1 year to 40.1% at 2 years and 43.3% at 3 years following index procedure.

**TABLE 2:** Relative risk (RR) associated with repeat treatment within 1 year following onabotulinumtoxinA injection.

Variable name	Basic statistics			Univariate model RR		Multivariate model RR	
	Total, *N* (%) 5694 (100.0)	Event, n (%) 1788 (31.4)	*p‐*value	Relative risk (RR, 95% CI)	Global *p*‐value	Relative risk (RR, 95% CI)	Global *p*‐value
Age in years							
65–74	2162 (38.0)	716 (33.1)	0.024	Ref.	0.022	Ref.	0.022
75–84	2620 (46.0)	815 (31.1)		0.94 (0.86–1.02)		0.94 (0.86–1.02)	
≥ 85	912 (16.0)	257 (28.2)		0.85 (0.75–0.96)		0.85 (0.75–0.96)	
Race							
White	5073 (89.1)	1599 (31.5)	0.582	Ref.	0.580	Ref.	0.500
Non‐white	621 (10.9)	189 (30.5)		0.97 (0.85–1.09)		0.96 (0.84–1.09)	
Charlson comorbidity index							
0	1835 (32.2)	579 (31.6)	0.629	Ref.	0.625	Ref.	0.413
1–3	2689 (47.2)	855 (31.8)		1.01 (0.92–1.10)		0.99 (0.90–1.08)	
≥ 4	1170 (20.6)	354 (30.3)		0.96 (0.86–1.07)		0.93 (0.82–1.05)	
Claims‐based frailty index							
Not frail (CFI < 0.15)	1304 (22.9)	393 (30.1)	0.422	Ref.	0.421	Ref.	0.157
Prefrail (0.15 ≤ CFI < 0.25)	3211 (56.4)	1011 (31.50)		1.04 (0.95–1.15)		1.08 (0.98–1.19)	
Mildly to severely frail (CFI ≥ 0.25)	1179 (20.7)	384 (32.6)		1.08 (0.96–1.21)		1.13 (0.99–1.28)	
Area deprivation index national quartile							
Q1 (ADI 1–29)	1613 (28.4)	515 (31.9)	0.074	Ref.	0.075	Ref.	0.089
Q2 (ADI 32 ≤ 48)	1397 (24.6)	425 (30.4)		0.95 (0.86–1.06)		0.96 (0.86–1.06)	
Q3 (ADI 50 ≤ 65)	1378 (24.2)	464 (33.7)		1.05 (0.95–1.17)		1.06 (0.95–1.17)	
Q4 (ADI ≥ 65)	1296 (22.8)	379 (29.2)		0.92 (0.82–1.02)		0.92 (0.82–1.03)	
Urodynamics in year prior to procedure							
No	3536 (62.1)	1054 (29.8)	0.001	Ref.	0.001	Ref.	0.003
Yes	2158 (37.9)	734 (34.0)		1.14 (1.06–1.23)		1.13 (1.04–1.22)	

*Note*: Beneficiaries who died within 1 year were excluded and model adjusted for procedure year.

Abbreviations: ADI = area deprivation index, CFI = claims‐based frailty index, CI = confidence interval, RR = relative risk).

Following SNM test procedures, 2725 (56.0%) of beneficiaries progressed to IPG placement, including 1890 (49.7%) of PNE procedures and 835 (78.6%) of Stage 1 procedures. Of the beneficiaries who ultimately progressed to IPG placement after Stage 1 test procedure, 209 (25%) had previously attempted PNE. Table 3 depicts results of the multivariable model for progression to IPG placement. Male beneficiaries who underwent Stage 1 procedures compared to PNE (aRR 1.57, 95% CI 1.50–1.64) were more likely to progress to IPG placement. Beneficiaries in ADI Q4 (lowest socioeconomic status) compared to ADI Q1 (aRR 1.10, 95% CI 1.03–1.18) were also more likely to progress to IPG placement. Non‐White male beneficiaries were less likely to progress to IPG placement compared to White beneficiaries (aRR 0.88, 95% CI 0.81–0.97). Age, CCI, CFI and pre‐procedure urodynamics testing were not significantly associated with IPG placement.

**TABLE 3:** Relative risk associated with implantable pulse generator placement within 90 days of sacral neuromodulation test procedure.

Variable name	Basic statistics			Univariate model RR		Multivariate model RR	
	Total, *N* (%) *N* = 4865 (100.0)	Event, n (%) 2725 (56.0)	p‐value	Relative risk (RR, 95% CI)	*p*‐value	RR, 95% CI	*p*‐value
Index procedure							
Percutaneous nerve evaluation	3802 (78.2)	1890 (49.7)	<0.001	Ref.	<0.001	Ref.	<0.001
Stage 1	1063 (21.9)	835 (78.6)		1.58 (1.51–1.65)		1.57 (1.50–1.64)	
Age in years							
65–74	2005 (41.2)	1171 (58.4)	0.010	Ref.	0.010	Ref.	0.051
75–84	2225 (45.7)	1222 (55.0)		0.94 (0.89–0.99)		0.95 (0.90–1.00)	
≥ 85	635 (13.1)	332 (52.3)		0.90 (0.82–0.97)		0.92 (0.85–1.00)	
Race							
White	4372 (89.9)	2480 (56.7)	0.003	Ref.	0.003	Ref.	0.006
Non‐white	493 (10.1)	245 (49.7)		0.88 (0.80–0.96)		0.88 (0.81–0.97)	
Charlson comorbidity index							
0	1806 (37.1)	961 (53.2)	0.009	Ref.	0.010	Ref.	0.704
1–3	2245 (46.2)	1289 (57.4)		1.08 (1.02–1.14)		1.02 (0.97–1.08)	
≥ 4	814 (16.7)	475 (58.4)		1.10 (1.02–1.18)		1.03 (0.95–1.11)	
Claims‐based frailty index							
Not frail (CFI < 0.15)	1152 (23.7)	611 (53.0)	0.057	Ref.	0.058	Ref.	0.252
Prefrail (0.15 ≤ CFI < 0.25)	2809 (57.7)	1592 (56.7)		1.07 (1.00–1.14)		1.05 (0.99–1.12)	
Mildly to severely frail (CFI ≥ 0.25)	904 (18.6)	522 (57.7)		1.09 (1.01–1.18)		1.06 (0.98–1.16)	
Area deprivation index national quartile							
Q1 (ADI 1–29)	1086 (22.4)	587 (54.1)	0.048	Ref.	0.046	Ref.	0.018
Q2 (ADI 32 ≤ 48)	1244 (25.6)	679 (54.6)		1.01 (0.94–1.09)		1.01 (0.94–1.09)	
Q3 (ADI 50 ≤ 65)	1220 (25.1)	680 (55.7)		1.03 (0.96–1.11)		1.05 (0.98–1.13)	
Q4 (ADI ≥ 65)	1309 (26.9)	774 (59.1)		1.09 (1.02–1.17)		1.10 (1.03–1.18)	
Urodynamics in year prior to procedure							
No	2737 (56.3)	1529 (55.9)	0.870	Ref.	0.813	Ref.	0.622
Yes	2128 (43.7)	1196 (56.2)		1.01 (0.96–1.06)		0.99 (0.94–1.04)	

*Note*: Beneficiaries who died within 1 year were excluded and model adjusted for procedure year.

Abbreviations: ADI = area deprivation index, CFI = claims‐based frailty index, CI = confidence interval, RR = relative risk.

**FIGURE 1:**
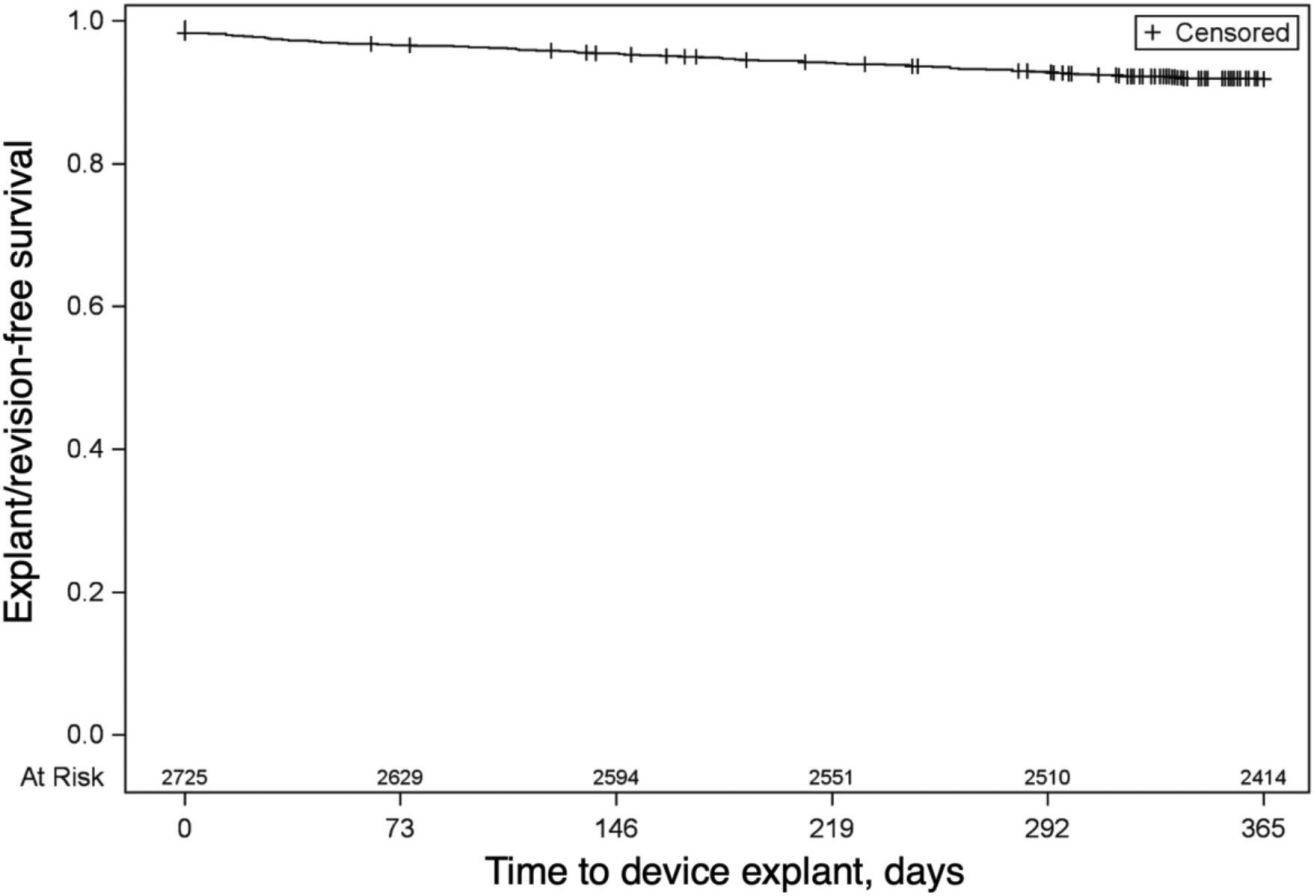
Kaplan–Meier estimate of sacral neuromodulation device explant or revision within 1 year of IPG placement.

Following IPG placement, 222 of 2725 (8.2%) of beneficiaries alive at 1 year underwent device explant or revision within 1 year (Figure 1). On multivariable analysis (Table S2), those who underwent Stage 1 versus PNE (aRR 2.0, 95% CI 1.56–2.57) were more likely to undergo device explant or revision. Conversely, beneficiaries with higher ADI quartiles (lower socioeconomic status) were less likely to undergo device explant or revision (aRR 0.65, 95% CI 0.45–0.93 for ADI Q3 vs. Q1 and aRR 0.67, 95% CI 0.47–0.95 for ADI Q4 vs. Q1). Age, race, CCI, CFI and pre‐procedure urodynamics testing were not significantly associated with device explant or revision.

Overall, 30‐day complications were experienced by 2305 (20.4%) of male beneficiaries, including 1339 (21.7%) who underwent onabotulinumtoxinA injections and 966 (18.9%) who underwent SNM test procedures (SMD = 0.071). Table S3 lists rates of individual complications. The most common type of complication for both minimally invasive OAB procedures was UTI, which was identified in 899 (14.6%) of onabotulinumtoxinA injection procedures and 438 (8.6%) of SNM test procedures (SMD = 0.189). One‐year mortality was higher among those who underwent onabotulinumtoxinA injections compared to SNM test procedures (7.6% vs. 5.0%, SMD = 0.109).

## 4 DISCUSSION

This study represents a large, nationally representative sample of older men who underwent intradetrusor onabotulinumtoxinA injections and SNM. During the study period, the number of male beneficiaries undergoing onabotulinumtoxinA injections increased while the absolute number undergoing SNM test procedures decreased. Additionally, the rate of repeat onabotulinumtoxinA injections tended to be lower than rates of progression from SNM test procedure to IPG implant: 31.6% of male beneficiaries who underwent onabotulinumtoxinA injections had a repeat injection within 1 year and 56.0% of those who underwent SNM test procedures progressed to IPG placement within 90 days. While older age was associated with lower relative risk of repeat onabotulinumtoxinA injections, beneficiary‐level factors such as frailty and CCI were not significantly associated with repeat onabotulinumtoxinA injections or IPG implantation. Additionally, nonclinical factors, such as race and socioeconomic status, were associated with IPG implantation following SNM test procedures, and the type of SNM test procedure (i.e. PNE vs. staged approach) was a predictor of both IPG placement and explant or revision. While direct comparisons between treatment modalities cannot be made because of differences in outcome measures, this study provides valuable insights into a population that is often under sampled in clinical trials.

This study demonstrates a steady increase in the number of onabotulinumtoxinA injections performed among male Medicare beneficiaries from 2014 to 2016 with a corresponding decrease in SNM test procedures. While this study focussed on a cohort who underwent treatment nearly 10 years ago, similar trends recently reported in a more contemporary cohort of commercially insured women,^21^ in which utilisation of onabotulinumtoxinA injections increased from 24% to 52% of all minimally invasive OAB procedures between 2013 and 2019 with a corresponding decrease in SNM utilisation. The present study redemonstrates these trends among older men who, like younger women, appeared to be preferentially receiving more onabotulinumtoxinA injections than SNM test procedures for OAB. Based on these data, it appears that clinicians gravitated away from SNM utilisation in favour of onabotulinumtoxinA injections following its regulatory approval in 2013.

Despite increasing use of onabotulinumtoxinA injections during the study period, the rate of repeat onabotulinumtoxinA injections among older male beneficiaries was lower than reported in previously published prospective trials that focus on female subjects. Compared to the ROSETTA trial, which included younger women (mean age 62.9 years), 2‐year rates of repeat onabotulinumtoxinA reported here among men were significantly lower (72% vs. 40%).^22^ These discrepancies may be explained by participants in the ROSETTA trial receiving relatively high onabotulinumtoxinA doses (200 U) in the setting of a controlled clinical trial. It is also possible that men, with greater urethral length and higher reported rates of incomplete urine emptying following onabotulinumtoxinA injections compared to women, are dissuaded from repeat injections because of procedure‐associated discomfort or adverse side effect profile.^23^ Diagnostic challenges in men with voiding dysfunction, including symptom overlap between BOO, detrusor underactivity and OAB, may also impact treatment adherence.^3^ Notably, men who underwent cystometrogram in the year prior to index onabotulinumtoxinA injection were more likely to undergo repeat injections within 1 year, suggesting possible diagnostic utility of urodynamics in men. Ultimately, prospective trials are needed to determine the efficacy of onabotulinumtoxinA injections for men with OAB.^3^

This study shows that 56% of men who undergo SNM test procedures will progress to IPG placement, including over 78% of men who underwent Stage 1. These rates of IPG implantation are similar to those reported in prior work examining similar outcomes. For example, rates of IPG implantation following SNM test procedures among Medicare beneficiaries (91.3% female) from 1997 to 2007 were 47.5% for beneficiaries undergoing PNE and 44.9% undergoing Stage 1, compared to 49.7% and 78.6%, respectively, in the present study.^24^ Compared to more contemporary series assessing patient response following IPG implantation, however, these rates of device implantation are lower than expected. For example, the ARTISAN‐SNM study (*N* = 129, 98% female) reported that 93% of participants experienced >50% reduction in urge incontinence episodes 2 years following single‐stage IPG implantation.^25^ While the ARTISAN‐SNM trial reports outcomes in a largely female population in the setting of a tightly controlled clinical trial, it is possible that advances in SNM technology or better patient selection explain the differences seen in treatment response rates reported in ARTISAN‐SNM and device implantation rates reported here.

Of those who underwent SNM test procedures, the majority underwent PNE versus Stage 1, and Stage 1 was associated with superior rates of IPG implantation compared to PNE. This observation has been previously documented^26^, ^27^ and is likely explained by PNE being less invasive than Stage 1 and thus more likely to be considered as a ‘trial’ procedure by patients who may be poor candidates for surgery. It is worth noting, however, that this persistence remains even after adjusting for patient‐specific factors, including age, comorbidity and frailty, consistent with prior work.^10^, ^27^ Similarly, the predominance of PNE versus Stage 1 test procedures likely reflects contemporary real‐world practice patterns on older Medicare beneficiaries, in whom a less invasive test procedure may be preferred. Ultimately, while progression from SNM test procedure to IPG implantation is a convenient and established surrogate for positive response to SNM test procedure,^27^, ^28^ patients may defer IPG implantation for a multitude of reasons other than inadequate symptom improvement which are not available in retrospective claims‐based analyses.

Frailty was not significantly associated with repeat onabotulinumtoxinA injections or progression to IPG implantation. Previously, our group has shown that increasing frailty portends worse outcomes following other invasive urologic procedures, such as surgery for bladder outlet obstruction and sling procedures for stress urinary incontinence.^14^ While it is possible that—in this real‐world sample—clinicians had selected out patients unable to undergo surgery, these findings do suggest that older men should not be excluded from onabotulinumtoxinA injections and SNM based solely on advanced age, comorbidity or frailty because of concerns of limited efficacy. However, consistent with prior literature, these patients may still be at a higher risk for perioperative complications. The rate of UTI's among beneficiaries who underwent onabotulinumtoxinA injections (14.6%), for example, is high. However, it is worth noting that these rates are comparable to those previously reported in smaller cohorts of men^29^ and lower than those reported in female populations.^22^ These findings underscore the importance of individualised perioperative risk assessment.^15^

The observation in this study that higher ADI (lower socioeconomic status) was associated with higher rates of IPG placement and lower risk of explant/revision is somewhat unexpected. The precise reason for this finding is unclear but may be related to unmeasured confounding factors, such as health‐care access or health literacy.^30^ While beneficiaries in lower SES areas may be less likely to be offered minimally invasive OAB therapy, those who undergo test procedures may be more likely to report symptom improvement or follow the advice of their treating clinician and subsequently agree to IPG placement. Interestingly, ADI was not associated with repeat onabotulinumtoxinA injections, perhaps because of the longer time interval from index to repeat injections compared to the conventional interval between SNM test procedure and IPG placement. Ultimately, there is a paucity of data on socioeconomic disparities associated with minimally invasive OAB therapy, and these findings suggest that social determinants of health may impact outcomes following SNM.

This study should be considered in the context of certain limitations. First, repeat onabotulinumtoxinA injections and progression to SNM implantation do not necessarily reflect treatment success or failure. Qualitative data, such as patient reported outcomes, as well as objective data, such as post‐void residual or urodynamic changes, are important to consider to distinguish treatment failure because of lack of symptom improvement from other factors, such as adverse events. Additionally, while baseline characteristics are not markedly different between the SNM or onabotulinumtoxinA groups, this is a retrospective study without randomisation or a control group, and therefore, clinician selection bias may impact results. Kaplan–Meier analysis was used to estimate cumulative incidence of repeat onabotulinumtoxinA injection rates at 2 and 3 years, but non‐informative censoring cannot be assumed in claims data. Therefore, losses to follow‐up may be related to either the treatment or outcome, which may bias results. This study lacks Part D (prescription drug) information, and thus, the dose of onabotulinumtoxinA used during injection, as well as concurrent or subsequent use of OAB pharmacotherapy, is not known. Therefore, onabotulinumtoxinA dosing patterns or dose–response relationships cannot be assessed. While beneficiaries undergoing SNM and onabotulinumtoxinA injections are likely to have similar rates of OAB pharmacotherapy utilisation, further study is warranted. Despite these known limitations, Medicare claims data are also a strength of this study in other ways, allowing us to analyse real‐world patient outcomes based on procedure codes in the largest reported cohort of men who underwent minimally invasive OAB therapy.

## 5 CONCLUSIONS

Despite increasing use of onabotulinumtoxinA injections among male Medicare beneficiaries from 2014 to 2016, men experience lower rates of repeat injections than rates previously reported among younger, predominantly female populations. Meanwhile, a similar proportion of men undergoing SNM test procedures will progress to IPG implantation compared to rates reported in cohorts of younger women. Clinical characteristics, such as comorbidity and frailty, were not associated with repeat onabotulinumtoxinA injections or IPG implantation; however, non‐clinical variables, such as lower socioeconomic status, were associated with higher rates of IPG implantation. Future studies should aim to assess primary drivers of minimally invasive therapy utilisation, as well as patient‐reported outcomes following minimally invasive OAB therapy in populations of men, who tend to be under‐sampled in OAB trials.

## AUTHOR CONTRIBUTIONS

**Leo D. Dreyfuss:** Conceptualization; methodology; manuscript creation. **Lufan Wang:** Conceptualization; statistical analysis; methods. **Farnoosh Nik‐Ahd:** Conceptualization; methodology; manuscript editing. **Abigail Shatkin‐Margolis:** Conceptualization; manuscript review. **Kenneth Covinsky:** Manuscript review. **W. John Boscardin:** Conceptualization; statistical analysis; manuscript editing. **Anne M. Suskind:** Conceptualization; methodology; supervision; manuscript editing.

## CONFLICT OF INTEREST STATEMENT

The authors have declared that no conflict of interest exists.

## Supporting information

Table S1: Current Procedural Terminology (CPT) and International Classification of Disease (ICD) codes used to define study cohort. ICD codes for overactive bladder adapted from Campbell et al, 2021.^10^
Table S2: Relative risk associated with implantable pulse generator (IPG) explant or revision within 1 year of IPG implant. Deaths within 1 year excluded, model adjusted for procedure year. (CI=Confidence Interval, RR = Relative Risk).
Table S3: Complications within 30‐days and 1‐year mortality following intravesical onabotulinumtoxinA or sacral neuromodulation (SNM) from 2014 to 2016. (SMD=Standard Mean Difference, UTI=Urinary Tract Infection, DVT/PE = Deep Vein Thrombosis/Pulmonary Embolus). Observations ≤10 suppressed per Centers for Medicare & Medicaid Services (CMS) cell‐suppression policy.^18^
