# Supplementary material for: Outcomes following intradetrusor onabotulinumtoxinA injections and sacral neuromodulation in older men
Source: BJUI Compass. 2026 Mar 31;7(4):e70168. doi: 10.1002/bco2.70168 (PMC13098358; doi:10.1002/bco2.70168)
Supplement: Supplementary file 1 — Table S1: Current Procedural Terminology (CPT) and International Classification of Disease (ICD) codes used to define study cohort. ICD codes for overactive bladder adapted from Campbell et al, 2021.10 Table S2: Relative risk associated with implantable pulse generator (IPG) explant or revision within 1 year of IPG implant. Deaths within 1 year excluded, model adjusted for procedure year. (CI=Confidence Interval, RR = Relative Risk). Table S3: Complications within 30‐days and 1‐year mortality following intravesical onabotulinumtoxinA or sacral neuromodulation (SNM) from 2014 to 2016. (SMD=Standard Mean Difference, UTI=Urinary Tract Infection, DVT/PE = Deep Vein Thrombosis/Pulmonary Embolus). Observations ≤10 suppressed per Centers for Medicare & Medicaid Services (CMS) cell‐suppression policy.18 [file BCO2-7-e70168-s001.docx]

Supplemental table 1: Current Procedural Terminology (CPT) and International Classification of Disease (ICD) codes used to define study cohort. ICD codes for overactive bladder adapted from Campbell et al, 2021 (10).

| **Procedure Codes** | **CPT-4** | |
| --- | --- | --- |
| Intradetrusor onabotulinumtoxinA injections | 52287 | |
| Percutaneous nerve evaluation (PNE) | 64561 | |
| Stage 1 permanent tined lead placement | 64581 | |
| Sacral Neuromodulation Device Implant | 64590 | |
| Sacral Neuromodulation Device Explant | 64585 | |
| Sacral Neuromodulation Device Revision | 64595 | |
| Simple Cystometrogram | 51725 | |
| Complex Cystometrogram | 51726 | |
| Complex Cystometrogram with Urethral Pressure Profile | 51727 | |
| Cystometrogram with Void pressure studies | 51728 | |
| Cystometrogram with Void pressure studies & UPP | 51729 | |
|  | | |
| **ICD Codes** | **ICD-9** | **ICD-10** |
| Hypertonicity of bladder/Overactive bladder | 596.51 | N3281 |
| Urge incontinence | 788.31 | N3941 |
| Urgency of urination | 788.63 | R3915 |
| Urinary frequency/Frequency of micturition | 788.41 | R35 |
| Mixed incontinence | 788.33 | N3946 |
| Nocturia | 788.43 | R351 |
| Enuresis/Enuresis not due to a substance/physiologic condition | 307.6 | F98 |
| Trigonitis/Trigonitis without hematuria | 595.3 | N303 |
| Trigonitis/Trigonitis with hematuria | 595.3 | N3031 |
| Infection of cystostomy/Urethral syndrome, unspecified | 597.81 | N3942 |
| Unspecified urinary incontinence | 788.3 | R32 |
| Incontinence without sensory awareness | 788.34 | N3942 |
| Post-void dribbling | 788.35 | N3943 |
| Nocturnal enuresis | 788.36 | N3944 |
| Continuous leakage | 788.37 | N3945 |
| Overflow incontinence | 788.38 | N3949 |
| Other urinary incontinence/Other specified urinary incontinence | 788.39 | N39498 |
| Polyuria/Other polyuria | 788.42 | N358 |
| Functional urinary incontinence | 788.91 | N3981 |

Supplemental Table 2: Relative risk associated with implantable pulse generator (IPG) explant or revision within 1 year of IPG implant. Deaths within 1 year excluded, model adjusted for procedure year. (CI=Confidence Interval, RR=Relative Risk).

|  | **Basic Statistics** | | | **Univariate Model RR** | | **Multivariate Model RR** | |
| --- | --- | --- | --- | --- | --- | --- | --- |
| **Variable Name** | **Total, N (%)**  N=2,725 (100.0) | **Event, n (%)** n=222 (8.2) | **P value** | **Relative risk**  **(HR, 95% CI)** | **P value** | **RR, 95% CI** | **P value** |
| **Procedure group** |  |  |  |  |  |  |  |
| Percutaneous Nerve Evaluation | 1,890 (69.4) | 115 (6.1) | <0.001 | Ref. | <0.001 | Ref. | <0.001 |
| Stage 1 | 835 (30.6) | 107 (12.8) |  | 2.11 (1.64 - 2.71) |  | 2.00 (1.56 - 2.57) |  |
| **Age** |  |  |  |  |  |  |  |
| 65-74 | 1,171 (43.0) | 106 (9.1) | 0.324 | Ref. | 0.333 | Ref. | 0.431 |
| 75-84 | 1,222 (44.8) | 91 (7.5) |  | 0.82 (0.63 - 1.08) |  | 0.84 (0.64 - 1.10) |  |
| ≥85 | 332 (12.2) | 25 (7.5) |  | 0.83 (0.55 - 1.26) |  | 0.87 (0.57 - 1.31) |  |
| **Race** |  |  |  |  |  |  |  |
| White | 2,480 (91.0) | 198 (8.0) | 0.323 | Ref. | 0.360 | Ref. | 0.312 |
| Non-white | 245 (9.0) | 24 (9.8) |  | 1.23 (0.82 - 1.84) |  | 1.25 (0.84 - 1.87) |  |
| **Charlson Comorbidity Index** |  |  |  |  |  |  |  |
| 0 | 961 (35.3) | 75 (7.8) | 0.887 | Ref. | 0.886 | Ref. | 0.944 |
| 1 – 3 | 1,289 (47.3) | 107 (8.3) |  | 1.06 (0.80 - 1.41) |  | 1.05 (0.78 - 1.42) |  |
| ≥4 | 475 (17.4) | 40 (8.4) |  | 1.08 (0.75 - 1.56) |  | 1.03 (0.70 - 1.53) |  |
| **Claims-based Frailty Index** |  |  |  |  |  |  |  |
| Not Frail (CFI<0.15) | 611 (22.4) | 51 (8.4) | 0.768 | Ref. | 0.775 | Ref. | 0.663 |
| Prefrail (0.15 ≤ CFI < 0.25) | 1,592 (58.4) | 125 (7.9) |  | 0.94 (0.69 - 1.29) |  | 0.92 (0.67 - 1.28) |  |
| Mildly To Severely Frail (CFI ≥ 0.25) | 522 (19.2) | 46 (8.8) |  | 1.06 (0.72 - 1.55) |  | 1.07 (0.71 - 1.60) |  |
| **Area Deprivation Index National Quartile** |  |  |  |  |  |  |  |
| Q1 (ADI 1 – 29) | 587 (21.6) | 63 (10.7) | 0.008 | Ref. | 0.011 | Ref. | 0.041 |
| Q2 (ADI 32 – < 48) | 679 (25.0) | 64 (9.4) |  | 0.88 (0.63 - 1.22) |  | 0.90 (0.65 - 1.25) |  |
| Q3 (ADI 50 – <65) | 680 (25.0) | 44 (6.5) |  | 0.60 (0.42 - 0.87) |  | 0.65 (0.45 - 0.93) |  |
| Q4 (ADI ≥ 65) | 774 (28.5) | 51 (6.6) |  | 0.61 (0.43 - 0.87) |  | 0.67 (0.47 - 0.95) |  |
| **Urodynamics in Year Prior to Procedure** |  |  |  |  |  |  |  |
| No | 1,529 (56.1) | 109 (7.1) | 0.028 | Ref. | 0.031 | Ref. | 0.129 |
| Yes | 1,196 (43.9) | 113 (9.5) |  | 1.33 (1.03 - 1.71) |  | 1.21 (0.95 - 1.56) |  |

Supplemental table 3: Complications within 30-days and 1-year mortality following intravesical onabotulinumtoxinA or sacral neuromodulation (SNM) from 2014-2016. (SMD=Standard Mean Difference, UTI=Urinary Tract Infection, DVT/PE=Deep Vein Thrombosis/Pulmonary Embolus). Observations ≤10 suppressed per Centers for Medicare & Medicaid Services (CMS) cell-suppression policy (18).

| **Variable name**  **N (%)** | **Total**  11,286 (100.0) | **OnabotulinumtoxinA**  6,166 (54.6) | **Sacral Neuromodulation**  5,120 (45.4) | **SMD*** |
| --- | --- | --- | --- | --- |
| **Number complications** |  |  |  |  |
| Any complication | 2,305 (20.4) | 1,339 (21.7) | 966 (18.9) | 0.071^†^ |
| 0 | 8,981 (79.6) | 4,827 (78.3) | 4,154 (81.1) | 0.086^†^ |
| 1-2 | 2,155 (19.1) | 1,237 (20.1) | 918 (17.9) |  |
| ≥3 | 150 (1.3) | 102 (1.7) | 48 (0.9) |  |
| **Complication type** |  |  |  |  |
| UTI | 1,337 (11.9) | 899 (14.6) | 438 (8.6) | 0.189^†^ |
| Cardiovascular | 605 (5.4) | 324 (5.3) | 281 (5.5) | 0.010 |
| Pulmonary | 303 (2.7) | 191 (3.1) | 112 (2.2) | 0.057^†^ |
| Acute renal failure | 233 (2.1) | 149 (2.4) | 84 (1.6) | 0.055^†^ |
| DVT/PE | 168 (1.5) | 100 (1.6) | 68 (1.3) | 0.024 |
| Reoperation | 155 (1.4) | 18 (0.3) | 137 (2.7) | 0.198^†^ |
| Other infection | 60 (0.5) | 42 (0.7) | 18 (0.4) | 0.046^†^ |
| Other complications | 46 (0.4) | 26 (0.4) | 20 (0.4) | 0.005 |
| Postoperative stroke | 37 (0.3) | 17 (0.3) | 20 (0.4) | 0.020 |
| Delirium | >29 (>0.2) | 22 (0.4) | <11 (<0.2) | 0.035 |
| Wound complication | >24 (>0.2) | <11 (<0.2) | <11 (<0.2) | 0.055^†^ |
| Postoperative hemorrhage | >14 (>0.1) | <11 (<0.2) | <11 (<0.2) | 0.012 |
| Postoperative shock | >6 (>0.1) | <11 (<0.2) | <11 (<0.2) | 0.017 |
| Anesthesia | <11 (<0.1) | <11 (<0.2) | <11 (<0.2) | 0.019 |
| **1-Year mortality** | 725 (6.4) | 470 (7.6) | 255 (5.0) | 0.109^†^ |

*SMD of 0.2 corresponds to 85% overlap between distributions, representing relative similarities between the two groups.

^†^Corresponding p-value <0.05
